# Mobile health interventions tailored to immigrant populations with diabetes: an integrative review

**DOI:** 10.1186/s12889-025-25241-3

**Published:** 2025-11-14

**Authors:** Jing Liu, Ora Z. Friedman, Ximin Yang, Haili Song, Mary Ann Sevick, Natalie Levy, Kosuke Tamura, Bei Wu, Lu Hu

**Affiliations:** 1 Center for Healthful Behavior Change, Institute for Excellence in Health Equity, NYU Grossman School of Medicine NYU Langone Health, 180 Madison Ave, New York, NY 10016 USA; 2 Department of Population Health, NYU Grossman School of Medicine, NYU Langone Health, New York, NY USA; 3 Department of Medicine, NYU Grossman School of Medicine, NYU Langone Health, New York, NY USA; 4 Socio-Spatial Determinants of Health Laboratory, Population and Community Health Sciences Branch, Division of Intramural Research, National Institute on Minority Health and Health Disparities, National Institutes of Health, Bethesda, MD USA; 5 NYU Shanghai, Shanghai, China

**Keywords:** Diabetes mellitus, Diabetes intervention, Health equity, Immigrants, Immigrant health, Intervention, MHealth, Mobile health, Review, Telemedicine, Telehealth

## Abstract

**Background:**

Immigrant populations face numerous barriers to accessing evidence-based diabetes interventions. Mobile health (mHealth) interventions are increasingly being used to support individuals in managing diabetes. This review aims to synthesize the available evidence on mHealth interventions specifically designed for immigrant populations with diabetes.

**Methods:**

An integrative review was conducted following Whittemore and Knafl’s methodology. Studies from the inception of PubMed, Web of Science, Cochrane Library, CINAHL Ultimate, Embase, and APA PsycInfo up to July 2024 were searched. The Mixed Methods Appraisal Tool was used to assess the quality of the included studies. A constant comparison strategy was employed for data analysis.

**Results:**

A total of seven studies met the inclusion criteria for this review, including five randomized controlled trials (RCTs: two fully powered RCTs and three pilot RCTs) and two pre-post single-arm pilot studies. All studies were conducted in the United States. The mHealth interventions were tailored to Korean, Chinese, Marshallese, Latinx, and South Asian immigrants. The sample sizes varied from 17 to 250. Evidence from the included studies is primarily limited by statistical power due to their pilot designs and small sample sizes. Despite this limitation, all studies demonstrated high feasibility and acceptability of mHealth interventions for diabetes management among these immigrant groups. Participants also reported high levels of satisfaction with mHealth interventions. The included studies consistently reported significant improvements in a range of health, psychosocial, and behavioral outcomes within the intervention groups, including hemoglobin A1C levels, body weight, blood glucose, total cholesterol, triglycerides, low-/high-density lipoprotein levels, and blood pressure; and self-efficacy, mental health status, diabetes knowledge, and quality of life; as well as physical activity, self-management, and dietary behaviors. However, when compared to control groups, the reported effectiveness of mHealth interventions on these outcomes was inconsistent.

**Conclusions:**

This review demonstrates the feasibility and acceptability of mHealth interventions for diabetes management among within immigrant populations. The findings suggest that these interventions may serve as a viable strategy to improve health, psychosocial, and behavioral outcomes. Future RCTs with larger sample sizes are needed to provide more robust evidence of the effectiveness of mHealth interventions. Importantly, this review highlights the scarcity of mHealth-related studies focused on immigrant populations with diabetes and calls for more research to examine how to best support this underserved group.

**Supplementary Information:**

The online version contains supplementary material available at 10.1186/s12889-025-25241-3.

## Background

Diabetes presents significant challenges to individuals, families, and healthcare systems. In 2022, it affected approximately 830 million people globally, and its prevalence is projected to rise rapidly [1, 2]. In the United States (US), it is estimated that roughly one in three adults will be diagnosed with diabetes during their lifetime [3].

The United Nations defines *immigrants* as those first-generation “who change their country of usual residence, including people who move across international borders, regardless of legal status, duration of stay, or reasons for migration” [4]. Compared to the general population, immigrant populations face a higher burden of diabetes [5–7], and are more likely to experience challenges in accessing diabetes care services [6, 8, 9]. These challenges include but are not limited to language, cultural, and financial barriers, immigration status, limited insurance coverage, and a shortage of culturally and linguistically competent healthcare services [6, 8, 9]. A recent analysis of data from the National Health and Nutrition Examination Survey in the US showed that foreign-born immigrants are at a higher risk of diabetes than US-born adults, with a greater age-standardized prevalence of diagnosed diabetes compared to the non-immigrant population (12.6% vs. 10.6%) [5]. A meta-analysis on the prevalence of type 2 diabetes in European countries showed that all foreign-born groups (e.g., South Asia, Sub-Saharan Africa, and the Middle East/North Africa) had significantly higher odds of developing type 2 diabetes compared to individuals born in the host countries [7]. Another study in the US indicates that, after adjusting for key sociodemographic factors, foreign-born immigrants have 54% higher odds of having undiagnosed diabetes compared to US-born individuals, suggesting that immigrants are more frequently overlooked in timely diabetes diagnosis and subsequent care [10]. As a result, immigrants are more likely to be disadvantaged in managing diabetes.

Mobile health (mHealth) interventions have been increasingly used to support disease diagnosis, treatment, education, and may improve health outcomes [11]. The World Health Organization defined *mHealth* as “medical and public health practices supported by mobile devices, such as mobile phones, patient monitoring devices, personal digital assistants, and other wireless devices” [12]. Research has shown that mHealth interventions effectively improve health outcomes for individuals with diabetes [13, 14]. For example, a recent study involving 221 participants with type 2 diabetes found that, compared to usual care, those who received a clinical pharmacist and health coach–delivered mHealth intervention reported a significant reduction in hemoglobin A1C levels at 12 months [13].

There have been several reviews evaluating the effectiveness of mHealth in individuals with diabetes [14–17]. These reviews consistently demonstrate that mHealth has the potential to improve diabetes outcomes [14–17]. Some also suggested that mHealth may serve as a strategy to reduce racial and ethnic disparities in access to diabetes care among underserved populations, such as African American and Hispanic individuals [16]. However, none of these reviews focused on immigrant populations, who face distinct challenges such as language, cultural, and financial barriers, lack of insurance coverage, and limited access to culturally and linguistically competent healthcare services [6, 8, 9]. Although studies have indicated that mHealth interventions hold promise for improving diabetes outcomes, their utilization and effectiveness in immigrant populations have not been reviewed.

This review thus aims to provide a comprehensive understanding of existing mHealth interventions tailored to immigrant populations with diabetes. The objectives of this review are to: (1) identify and describe the mHealth interventions specifically designed for immigrant populations with diabetes, and (2) assess the effectiveness of these interventions on diabetes outcomes in this population. The findings of this review have important implications for researchers, as immigrant groups, particularly those with limited English proficiency, are often excluded from mHealth trials due to language barriers or concerns about digital literacy. By addressing this critical gap in the literature, this review offers a timely and relevant summary of mHealth interventions for diabetes management specifically among immigrant populations.

## Methods

An integrative review was performed. Integrative reviews are particularly advantageous when researchers seek to gain a holistic understanding of the study topic [18], which aligns well with the aim of this study. The review process was guided by the five-step strategy proposed by Whittemore and Knafl [18].

### Literature search

The PICO model (Patient/Population, Intervention, Comparison, and Outcomes) was used to inform the literature search for this review [19]. The identification of these elements clarifies the scope of the review and ensures the quality of literature searches [19]. The PICO for the current review is presented in Table 1. Key search terms were developed based on the PICO of this review, including diabetes, diabetes mellitus, telemedicine, telehealth, e-health, eHealth, mHealth, m-health, mobile health, digital health, video, message, internet, migrant, and immigrant.

**Table 1:** The PICO of the current review

PICO elements	
Patient/Population	Immigrants aged 18 years or older with any type of diabetes
Intervention	Using mHealth approaches
Comparison	Any comparators, such as usual care or other face-to-face interventions
Outcomes	Any patient-centered outcomes related to diabetes care

#### Inclusion and exclusion criteria

The review included studies based on the following inclusion criteria: (1) participants were adults (18 years or older) diagnosed with type 1, type 2, or gestational diabetes, (2) the interventions were exclusively tailored for first-generation immigrant populations, as defined by the United Nations [4], (3) the interventions were delivered using mHealth approaches via mobile devices, as defined by the World Health Organization [12], (4) the study design could be an RCT, quantitative non-randomized study, quantitative descriptive study, qualitative study, mixed methods study or pilot study, and (5) the study was published in English.

Studies were excluded if: (1) the article only described the development or adaptation processes of mHealth interventions and did not provide any empirical results on participants’ diabetes outcomes after using the mHealth intervention, or (2) the study was a review, meta-analysis, case study, government report, conference paper, study protocols or trial registrations.

#### Information sources

We searched for studies from the inception of PubMed, Web of Science, Cochrane Library, CINAHL Ultimate, Embase, and APA PsycInfo up to July 2024. We also manually reviewed the reference lists of relevant studies. The information search was conducted in July 2024 and primarily carried out by the first author (JL).

#### Search strategy

The full search strategies in each database are provided in Additional File 1: Supplementary Table 1.

#### Selection process

**Fig. 1:**
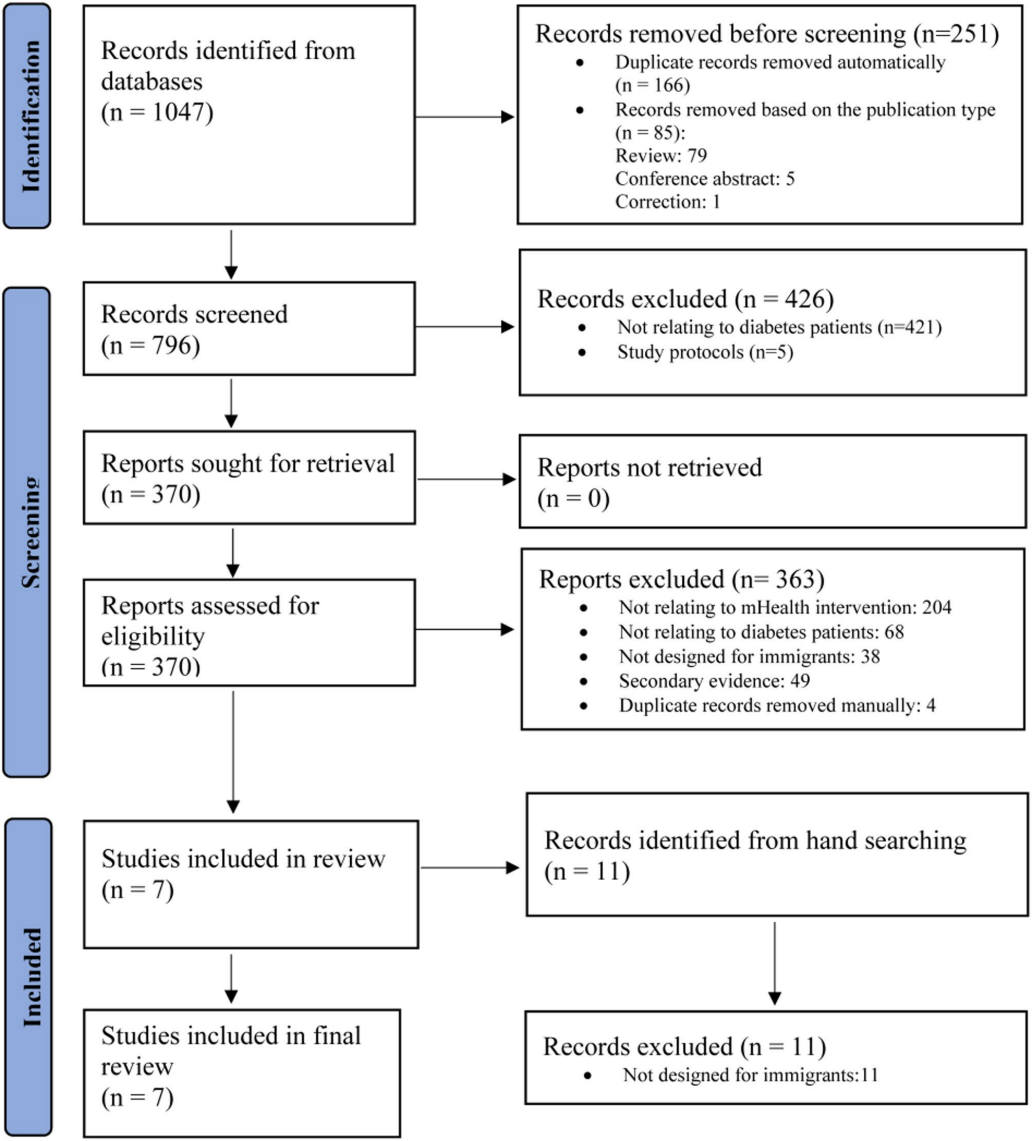
Study selection process

EndNote software was used for study selection. Duplicate studies were identified and removed using EndNote’s de-duplication feature. Titles and abstracts of the remaining studies were then screened based on the inclusion and exclusion criteria. Full-text articles were retrieved for further eligibility assessment. The study selection process was conducted by four authors (JL, OF, XY, and HS), with each step independently carried out by at least two authors to ensure reliability. Any disagreements during the selection process were discussed and resolved in regular research meetings involving all authors. A total of 1,047 studies were identified through database searches, and an additional 11 were identified through manual searching. However, only seven studies met the eligibility criteria and were included in the final analysis. The detailed selection process is illustrated in the PRISMA flow diagram (Fig. 1).

#### Data evaluation

The methodological quality of the included studies was appraised using the Mixed-Methods Appraisal Tool (MMAT) [20]. This tool was selected because it is designed to evaluate the methodological quality of a broad range of empirical evidence. It includes two screening questions and five appraisal criteria, specifically tailored to quantitative, qualitative, and mixed-methods research, making it suitable for this integrative review. In this review, each study was carefully reviewed to determine whether it satisfied the screening questions and relevant criteria, with responses recorded as “yes,” “no,” or “can’t tell.” Studies receiving a minimum of five “yes” responses were deemed high quality and included in the final analysis. Two independent reviewers conducted the methodological appraisal. The entire research team discussed and resolved discrepancies until a consensus was reached. Ultimately, the authors agreed that all seven studies should be included. A summary of the methodological quality of these studies is provided in Additional File 1: Supplementary Table 2.

#### Data analysis and presentation

A constant comparison strategy was employed for data analysis [18]. A review matrix was developed to facilitate data reduction and display. Studies were initially classified based on the study design. Data on the authors, year, country, sample, setting, intervention, comparison, outcomes, and key findings were identified. Data on the key features of each intervention were summarized. All data were counted, compared, and contrasted to identify similarities and differences among the studies. All authors reviewed the studies and engaged in discussions regarding conflicting results until a consensus was reached.

## Results

### Characteristics of the studies

A total of seven studies were included in the final analysis [21–27]. There were five RCTs (two fully powered RCTs and three pilot RCTs) [22, 23, 25–27], and two pre-post single-arm pilot studies [21, 24]. One study used qualitative interviews to collect follow-up data [24], while most used questionnaire surveys [21–23, 25–27]. All studies were conducted in the US and focused on type 2 diabetes. The sample sizes varied from 17 to 250. These immigrants had Korean [23, 27], Chinese [21, 22], Marshallese [24], Latinx [26], and South Asian backgrounds [25]. The characteristics of the included studies are described in Table 2.

**Table 2:** Characteristics of the included studies (*n* = 7)

Study & year & country	Design	Sample & setting	Intervention	Comparison	Timing of measurement	Outcomes	Key findings
Kim 2009 [23] US	RCT (Pilot)	79 Korean immigrants with uncontrolled type 2 diabetes Intervention: 40 Control: 39 Korean communities in theBaltimore-Washington area	A three-component intervention: (1) psychobehavioral education, (2) home glucose monitoring with tele-transmission, and (3) monthly individualized telephone counseling	Usual care (delayed intervention)	Baseline, 18, 30 weeks	• A1C • Fasting glucose • Lipid panel • Blood pressure • Weight • Diabetes knowledge • Self-efficacy • Self-care activities • Depression • Quality of life	• Retention rate: 95.2% • Participation rates: 83%–94% for each education session. • Satisfaction: mean score of 9.7 (out of 10) • Outcomes significantly improved by intervention compared to control group: A1C (at all measurement times), fasting glucose (at 18 weeks but not at 30 weeks), total cholesterol (at 30 weeks), triglycerides (at 30 weeks), diabetes knowledge (at all measurement times), self-care activities(at all measurement times), self-efficacy (at all measurement times), attitudes toward diabetes (at all measurement times), depression (at 18 weeks but not at 30 weeks), and quality of life (at all measurement times) • Outcomes with no significant difference between groups: body mass index, systolic blood pressure, diastolic blood pressure, HDL, and LDL
Kim 2015 [27] US	RCT	250 Korean immigrants with type 2 diabetes Intervention: 120 Control: 130 Korean communities in theBaltimore-Washington area	A three-component intervention: (1) a series of structured behavioral education programs delivered in a group format, (2) ongoing self-monitoring of glucose, and (3) monthly individualized telephone counseling	A brief educational brochure	Baseline, 3, 6, 9, 12 months	• A1C • Blood glucose • Total cholesterol • Lipid panel • Quality of life • Self-efficacy • Adherence to diabetes management regimen • Health literacy	• Retention rate: 83.6% • Average participation rate: 96.1% • Outcomes significantly improved by intervention compared to the control group: A1C (at all measurement times), blood glucose (at all measurement times), LDL (at all measurement times), self-efficacy (at all measurement times), diabetes knowledge (at all measurement times), attitudes toward diabetes (at all measurement times), quality of life (at all measurement times), and depression (at 3 months) • Outcomes with no significant difference between groups: systolic blood pressure, diastolic blood pressure, triglycerides, HDL and total cholesterol
McElfish, 2019 [24] US	Pre-post study (pilot) Qualitative interviews were conducted at follow-up.	50 Marshallese immigrants with type 2 diabetesattending the pre-post intervention survey, 20 attending the follow-up interview A diabetes clinic in northwest Arkansas	A YouTube video-based educational intervention providing knowledge on how to use a glucometer to check blood glucose	Not applicable	Baseline, between 12 and 17 weeks	• Self-efficacy • A1C • Cultural appropriateness	• Cultural appropriateness: Most found the content clear, and all agreed that the intervention was culturally appropriate and effective. All participants learned how to monitor blood glucose and use a glucometer effectively through the intervention. • Outcomes significantly improved from pre- to post-intervention: self-efficacy and A1C
Rechenberg, 2021 [26] US	RCT (Pilot)	17 Latinx immigrants with type 2 diabetes and limited English proficiency Intervention: 10 Control: 7 Federally qualified health centers	A telephone-based, language-concordant health coaching intervention	Usual care and a written educational material in Spanish.	Baseline, 6 months	• A1C • Anxiety • Depression • Patient satisfaction	• Response rate: 82% • Satisfaction: mean score of 82.9 (out of 100) • Outcomes significantly improved from pre- to post-intervention: A1C, depression, and anxiety. No significant changes were observed in the control group. The study did not report between-group comparisons.
Hu 2022 [21] US	Pre-post study (pilot)	30 Chinese immigrants with type 2 diabetes New York City	Social media-based videos and biweekly telephone counseling	Not applicable	Baseline, 3, 6 months	• Feasibility • Acceptability • A1C • Self-efficacy • Dietary intake • Physical activity	• Retention rate: 100% at 3 months, 97% at 6 months. • Mean video watch rate: 92%. • Satisfaction: mean score of 9.9 (out of 10) • Outcomes significantly improved from pre- to post-intervention: A1C (at 6 months), self-efficacy (at 3 and 6 months), dietary intake (at 6 months), and physical activity (at 6 months)
Hu 2024 [22] US	RCT (pilot)	23 dyads (low-income Chinese immigrants with type 2 diabetes and their families/friends) Intervention: 11 Control: 12 New York City	24 educational videos with biweekly telephone counseling	Usual care	Baseline, 3, 6 months	• Feasibility • Acceptability • A1C • Weight • Dietary intake • Physical activity • Self-efficacy • Diabetes self-management behaviors • Diabetes distress • Support	• Retention rate: 90.91% • Average video watch rate: 76.8% (65%- 90%) • Satisfaction: mean score of 9.4 (out of 10) • Outcomes significantly improved from pre- to post-intervention but did not show significant improvement compared to the control group: A1C, body weight, sugary drink intake, and self-management, and emotional support • Outcomes with no significant improvements within or between groups: physical activity, self-efficacy, diabetes-specific support, and diabetes distress
Shah 2024 [25] US	RCT	190 South Asian immigrants with type 2 diabetes and comorbid hypertension Intervention: 97 Control: 93 South Asian community in Atlanta	A telehealth intervention consisting of 5 educational sessions and monthly telephone counseling	The control group received only the first education session.	Baseline, 6 months	• Feasibility • Blood pressure control • A1C • Weight • Body mass index • Physical activity • Daily diet intake • Medication adherence • Diabetes self-management • Physician management • Health self-efficacy • Depression risk • Instrumental support • Days of poor physical and mental health	• Feasibility: 94% completed all 5 sessions, the action plan (98%), three or more progress notes (93%), both one-on-ones (94%), and the follow-up survey (95%) • Outcomes significantly improved by the intervention: body weight, BMI, systolic blood pressure, diastolic blood pressure, physical activity, medication adherence, daily fruit intake managing diabetes with diet control, mean days of poor physical health, and mean days of poor mental health • Outcomes significantly improved within intervention group, but the intervention effects were not significant: blood pressure control, checking feet daily, managing diabetes with physical activity, and sugar sweetened beverage intake • A1C was available for a small subset. A non-significant decrease was observed for the treatment group, but the intervention effect was not significant.

*RCT* Randomized Controlled Trials, *HDL* high-density lipoprotein, *LDL* low-density lipoprotein

### mHealth interventions

The key features of mHealth interventions are summarized in Table 3. The most frequently used mHealth technology was telephone counseling [21–23, 25–27]. Some studies also used videos and Zoom to provide diabetes management education to participants [21, 22, 24, 25]. One study used a tele-transmission system to provide remote glucose monitoring [23]. Briefly, Kim et al. (2009) developed and pilot-tested a culturally tailored mHealth intervention for Korean immigrants with type 2 diabetes (*n* = 79; 30-week follow-up), incorporating remote glucose monitoring, nurse counseling, and group education [23]. In 2015, they reported an RCT with a larger sample and a longer follow-up period (*n* = 250; 12-month follow-up) to evaluate the intervention’s efficacy [27]. McElfish et al. developed a YouTube video-based intervention focused on glucose monitoring for Marshallese immigrants (*n* = 50; 12–17-week follow-up) [24]. Rechenberg et al. piloted a telephone-based health coaching intervention for 17 Latinx immigrants with limited English proficiency, with a 6-month follow-up [26]. Hu et al. piloted a WeChat-based video intervention combined with telephone counseling for low-income Chinese immigrants with type 2 diabetes (*n* = 30; 6-month follow-up) [21], later refining the intervention to include family sessions and evaluating its impact on diabetes outcomes with 23 dyads (6-month follow-up) [22]. Shah et al. conducted an RCT to assess a Zoom-based educational program and telephone calls among 190 South Asian immigrants with type 2 diabetes and hypertension (6-month follow-up) [25].

**Table 3:** Summary of the mHealth interventions tailored for immigrants (*n* = 7)

Study	Device	Medium of communication	Component/content	Frequency and duration	Follow-up period
Kim 2009 [23]	Telephone	1) A study-specific tele-transmission system 2) Telephone calls (combined with conventional group education sessions)	1) The study-specific tele-transmission system was used for remote glucose monitoring. 2) Nurses performed regular telephone counseling with participants to reinforce diabetes knowledge, guide self-care, and provide emotional support.	1) Tele-transmission system: 24 weeks 2) Telephone counseling: monthly for 24 weeks	30 weeks
Kim 2015 [27]	Telephone	Telephone calls (combined with conventional group education sessions)	1) Group education sessions covering knowledge of diabetes, coping and enabling capacities, cognitive reframing, belief in self, positive perspective, and health literacy 2) Patient self-monitoring of glucose using a glucose monitor, strips, and lancets with the guidance of a nutritionist 3) Telephone counseling to assist them in reaching individualized treatment goals and maintaining acquired self-care skills and a healthy lifestyle	1) Group education sessions: weekly 2-hour sessions over the course of 6 weeks 2) Self-monitoring of glucose: twice a day for 12 months 3) Telephone counseling: once a month for 12 months	12 months
McElfish 2019 [24]	Not reported	YouTube videos	Researchers used a YouTube video to deliver patient education materials on how to use a glucometer to check blood glucose, what the numbers on the glucometer mean, and the information healthcare providers need about blood glucose check results, how diet and exercise can affect blood glucose levels, and what to do if blood glucose levels are too high or too low.	One-time video with a length of roughly 3 minutes	12–17 weeks
Rechenberg 2021 [26]	Telephone	Telephone calls	The coaching calls were of an emergent nature, allowing participants to simultaneously ask questions and discuss barriers and facilitators of diabetes management. Patients and coaches also set goals together during each call.	Average five telephone coaching sessions every two weeks. Each call has an average duration of 18.33minutes.	6 months
Hu 2022 [21]	Telephone	1) WeChat videos 2) Telephone calls	Researchers regularly delivered WeChat videos containing diabetes knowledge and behavioral change guidance in Mandarin Chinese. Researchers also called participants every 2 weeks to discuss the barriers and facilitators of diabetes management.	1) WeChat videos: 2 each week for a duration of 12 weeks. Each video lasts 5 minutes 2) Telephone calls: every 2 weeks for 12 weeks Each call lasted 15 minutes.	6 months
Hu 2024 [22]	Telephone	1) WeChat videos 2) Telephone calls	Researchers regularly delivered WeChat videos containing diabetes knowledge, behavioral change guidance and family orientation sessions in Mandarin Chinese. Researchers also called participants every 2 weeks to set goals, monitor progress, and discuss the barriers and facilitators of diabetes management.	1) WeChat videos: 2 each week for a duration of 12 weeks. Each video lasts 5–10 minutes. 2) Telephone calls: every 2 weeks for 12 weeks	6 months
Shah 2024 [25]	Telephone	1) Zoom-based education sessions 2) Telephone calls	Researchers conducted monthly patient education on type 2 diabetes, hypertension, nutrition, physical activity, stress management, and self-management via Zoom. Participants were asked to create an action plan for eating a healthy diet and being physically active. Researchers made monthly telephone calls to monitor progress and provide support.	1) Zoom-based education sessions: 5 sessions over a length of 6 months. Each session lasted 60 minutes. 2) Telephone calls: Monthly for 6 months. Each call lasted 30 minutes.	6 months

#### Theories and conceptual frameworks

Four studies reported the theories and conceptual frameworks used to develop their mHealth interventions [21, 22, 25, 27]. Hu et al. used the Social Cognitive Theory to guide the development of 24 culturally and linguistically tailored diabetes videos for Chinese immigrants with type 2 diabetes [21]. Building on this work, Hu et al. used the Individual and Family Self-Management Theory to adapt this patient-centered intervention into a family-oriented intervention, by including context, process, and outcome factors that may influence patient and family self-management behaviors [22]. Shah et al. used the principles of community-based participatory research, the Health Belief Model, and the Social Support Theory to support designing a community health worker-led type 2 diabetes management program for South Asian immigrants with diabetes [25]. Additionally, Kim et al. employed the PRECEDE model (Predisposing, Reinforcing, and Enabling Constructs in Education/Environmental Diagnosis and Evaluation) along with Braden’s Self-Help Model to inform the design of the intervention [27].

#### Devices and mediums

Telephones were employed as the primary mHealth devices in six studies, although the studies did not specify the type of telephone used (e.g., smartphone or traditional cellphone) [21–23, 25–27], whereas one study did not specify the device used [24]. The mediums of communication included telephone calls [21–23, 25–27], YouTube [24], social media platforms such as WeChat [21, 22], and Zoom [25] applications.

#### Components, duration and follow-up

All seven studies described the components of the interventions. These interventions primarily provided immigrants with culturally and linguistically tailored educational materials on diabetes knowledge and self-management techniques related to healthy eating, physical activity, medication adherence, or blood glucose self-monitoring skills. These interventions also incorporated goal setting, behavioral change, and remote glucose monitoring functions. The duration of these interventions varied from a one-time video educational session lasting roughly 3 minutes [24] to ongoing interactions lasting 12 months [27]. Follow-up occurred between 12 weeks [24] and 12 months [27] after the start of the intervention.

#### Cultural and linguistic consideration

All studies incorporated the distinct cultural and linguistic needs of immigrants with diabetes into the mHealth interventions [21–27]. Measures included involving researchers with similar cultural backgrounds to guide intervention design, integrating culturally relevant examples of diet and physical activities, and employing bilingual researchers to deliver education and consultations in participants’ native languages.

####  Feasibility and acceptability 

All studies reported high feasibility and acceptability, and most participants reported satisfaction with the studied mHealth intervention [21–27]. For example, Kim et al. reported participation rates ranging from 83% to 94%, a retention rate of 95.2%, and an overall satisfaction score of 9.7 (out of 10) with the education sessions among Korean immigrants. Most participants completed all mHealth intervention sessions [23]. Another study asked Marshallese immigrants about their experiences and found that most participants believed the video content was not confusing. All participants in this study affirmed that the intervention was culturally appropriate and effective [24].

#### Outcomes examined

A range of outcomes was examined in the included studies and was narratively described and categorized into three groups: (1) health outcomes, including hemoglobin A1C levels, body weight, blood glucose, total cholesterol, triglycerides, low-/high-density lipoprotein levels and blood pressure; (2) psychosocial outcomes, including self-efficacy, mental health status, diabetes knowledge, and quality of life; as well as (3) behavioral outcomes, including physical activity, self-management, and dietary behaviors.

##### Health outcomes

###### Hemoglobin A1C

Hemoglobin A1C was the most examined outcome, with all seven studies reporting the effectiveness of mHealth interventions in improving A1C levels among immigrants. Six of these studies indicated a significant reduction in hemoglobin A1C levels following the intervention compared to baseline levels within the intervention group [21–24, 26, 27]. Only one study reported a decrease that was not statistically significant, which may be due to the limited availability of A1C data, as it was only available for a small subset of the sample [25]. However, when compared to the control groups, the results were mixed. Several studies found no significant differences between the intervention group and the control group (two out of five; two studies were excluded due to single-arm designs, and one study did not compare outcomes between groups) [22, 25] (all *p* >0.05). In contrast, two studies among Korean immigrants (one pilot RCT and one fully powered RCT) reported a significant between-group difference in hemoglobin A1C levels at some measurement points [23, 27]. For example, in the pilot study, the A1c level in the intervention group improved significantly, decreasing by 1.2% (SD = 1.3%) at 18 weeks and 1.3% (SD = 1.3%) at 30 weeks. In comparison, the control group showed a hemoglobin A1C increase of 0.1% (SD = 1.7%) at 18 weeks and a reduction of 0.4% (SD = 1.4%) at 30 weeks [23].

###### **Body weight**

Two studies reported that the mHealth intervention may be effective in reducing body weight [22, 25]. In a pilot RCT study, Hu et al. found a trend toward a decline in participants’ body weight at 6 months in both the intervention (*n* = 11) and control (*n* = 12) groups. However, the reduction in body weight observed post-intervention, and the difference in body weight change between the intervention and control groups were not statistically significant (change = −11.7 lbs, 95% CI [−24.7, 1.4], *p* = 0.079; difference in change = 6.9 lbs, 95% CI [−25.28, 11.43], *p* = 0.459), which may be due to the pilot nature of the study and small sample size [22]. In contrast, in a fully powered RCT study, Shah et al. reported a significant reduction in mean body weight following the intervention (−4.8 [−8.2, −1.4], *p* = 0.006), as well as a significant decrease in body weight change in the intervention group (*n* = 97) compared to the control group (*n* = 93, adjusted effect = −5.2 [−9.0, −1.4], *p* = 0.007) [25].

###### **Blood glucose**

Two studies conducted by Kim and colleagues investigated blood glucose levels among Korean immigrants. In the earlier pilot study (*n* = 79), participants in the intervention group experienced a significant reduction in fasting glucose at 18 weeks compared to the control group (intervention group: change = −42.2 [66.6] mg/dL vs. control group: change = 0.8 [66.4] mg/dL, *p* = 0.01). However, this difference was not statistically significant at 30 weeks (−42.3 [61.6] mg/dL vs. −7.0 [57.2] mg/dL, *p* = 0.06) [23]. In the subsequent RCT (*n* = 250), while significant reductions in blood glucose levels were seen in both the intervention and control groups, the glucose levels in the intervention group were significantly lower than those in the control group throughout the 12-month follow-up period (difference = −15.0 [6.8] mg/dL, −24.0 [6.9] mg/dL, −17.3 [6.7] mg/dL, and −22.3 [6.6] mg/dL at 3, 6, 9, and 12 months, respectively; all *p* < 0.05) [27].

###### Other health outcomes

Two studies investigated total cholesterol, triglycerides, and low-/high-density lipoprotein levels [23, 27]. They reported improvements in these outcomes for the intervention groups at the measurement endpoints, yet the findings on total cholesterol were inconsistent when compared to the control groups [23, 27]. Both studies showed no significant improvement in low-/high-density lipoprotein levels by the intervention compared to the control groups [23, 27]. Besides, Shah et al. reported blood pressure control and levels as outcomes in their study of South Asian immigrants with both diabetes and uncontrolled hypertension: both systolic and diastolic blood pressure levels showed significant improvement in the intervention group when compared to the control group [25]. However, in this study, both the intervention and control groups showed improved blood pressure control (<130/80 mmHg), but the differences between them were not significant [25].

##### Psychosocial outcomes

###### Self-efficacy

Six studies examined the effectiveness of the mHealth intervention on self-efficacy, consistently reporting a significant increase from baseline to post-intervention in the self-efficacy scores [21–25, 27]. In Kim et al., among Korean immigrants, the intervention group (*n* = 40) showed a significant improvement in self-efficacy compared to the control group (*n* = 39, intervention group: change = 6.6 [14.4] vs. control group: change = −0.9 [15.1], *p* = 0.01) [23]. Kim’s team confirmed these results in a larger-scale RCT involving 250 Korean immigrants [27]. However, another study by Hu et al. found no significant difference in the changes in self-efficacy between the groups (intervention: *n* = 11, control: *n* = 12, Chinese immigrants, *p* = 0.952) [22]. Besides, in Shah et al., the difference in self-efficacy change between the intervention and control groups was not statistically significant (*p* = 0.762) [25].

###### Mental health status

Five studies reported the effectiveness of the mHealth intervention on mental health outcomes [22, 23, 25–27]. These studies consistently reported significant within-group improvements in depression [23, 25–27], anxiety [26], and diabetes stress [22], and days of poor mental health [25] when compared to the baseline levels. However, when compared to the control groups, three studies found a significant improvement in depression [23, 27] or days of poor mental health [25] in the intervention group at some measurement points, while two other pilot studies did not report significant between-group differences [22, 26].

###### Diabetes knowledge

Three studies assessed the effectiveness of the mHealth intervention on diabetes knowledge among immigrant populations, with all demonstrating promising outcomes [23, 24, 27]. Kim et al. reported that, compared to the control group, the intervention group showed significant improvements in diabetes knowledge at both 18 weeks (intervention: change = 2.2 [2.4] vs. control: change = 0.1 [3.2], *p* < 0.00) and 30 weeks (intervention: change = 2.4 [2.3] vs. control: change = 0.7 [2.4], *p* < 0.00) following intervention [23]. The subsequent RCT by Kim et al. in 2015 confirmed these findings, reporting statistically significant improvements in diabetes knowledge over the 12-month intervention period compared to the control group [27]. McElfish et al. reported that all participants indicated that they gained knowledge about how to monitor or control blood glucose and properly use a glucometer through the intervention [24].

###### Quality of life

Two studies conducted by Kim’s team involving Korean immigrants reported that, compared to the control group, the intervention group demonstrated significant improvements in quality of life at all measurement endpoints (all *p* < 0.05) [23, 27].

##### Behavioral outcomes

###### Physical activity

Three studies examined the effectiveness of mHealth interventions on physical activity among immigrant populations [21, 22, 25]. Two of these studies reported a significant increase in physical activity 6 months post-intervention compared to baseline levels [21, 25]. The changes in physical activity compared to the control group were significantly different in one study [25]. In another study, the results indicated no significant improvements in physical activity, both within and between groups, following the mHealth intervention [22].

###### Self-management

Two pilot studies reported the effectiveness of the mHealth intervention on self-management among immigrants and found significant improvements in self-management scores after the intervention compared to baseline levels [22, 23]. In Kim et al. [23], the intervention group showed significant improvements in self-care activities compared to the control group (*n* = 79, Korean immigrants). However, Hu et al. [22] found no significant difference in the change in self-management between the groups (*n* = 23, Chinese immigrants).

###### Dietary behavior

Two pilot studies conducted by Hu and colleagues explored the effectiveness of mHealth interventions on dietary behavior among Chinese immigrants [21, 22]. One demonstrated a significant improvement in dietary behavior at 6 months following the intervention, even though the difference was not statistically significant 3 months after the intervention [21]. In the other study, participants also showed a significant reduction in sugary drink intake 6 months following the intervention; however, this change did not significantly differ from the control group [22].

## Discussion

This integrative review aimed to synthesize the evidence on mHealth interventions specifically tailored to immigrant populations with diabetes. The review only identified seven studies: five RCTs and two pre-post single-arm studies. Only two of the seven studies had adequate statistical power, while the other five were pilot studies. This suggests a significant gap in evidence and limited efforts to explore these interventions within this marginalized group. Notably, all studies were conducted in the US. Other countries, such as those in Europe, Australia and Canada, have also been witnessing a rapid increase in their immigrant populations [28, 29]. Given that this group is at a higher risk for diabetes and faces greater barriers to accessing diabetes care services in their host countries [6, 8, 9], this review underscores the urgent need for increased research and initiatives aimed at developing and promoting mHealth interventions tailored to immigrant communities on a global scale.

This review found that mHealth interventions are both feasible and acceptable for immigrant populations to manage their diabetes. The reviewed studies consistently reported satisfactory participation rates, retention rates, and high satisfaction levels with the mHealth intervention. This finding aligns with other reviews indicating that mHealth receives positive feedback from other underserved groups in managing various health conditions, such as in ethnic minority [30] and Indigenous groups [31]. This finding suggests that culturally and linguistically relevant mHealth designs resonate well with immigrants and have the potential to bridge diabetes care gaps within this underserved group. This finding also reinforces the recognition by the World Health Organization that mHealth has the potential to enhance and support the digital landscape of healthcare delivery and promote the achievement of universal health coverage for all people [32].

This review found that mHealth interventions are promising for improving health outcomes among immigrants with type 2 diabetes, including hemoglobin A1C, body weight, and blood glucose. Although studies found notable improvements in hemoglobin A1C, body weight, and blood glucose control compared to baseline measurements, most indicated that the effectiveness of the interventions on these outcomes was not statistically significant compared to the usual care group. This may be mainly attributed to the fact that most of the included studies were at the pilot stages [21–24, 26]; the researchers of these studies consistently noted that the small sample sizes limited the statistical power to draw definitive conclusions. Therefore, future well-designed RCTs with larger sample sizes are clearly necessary to establish adequate statistical power and provide more robust evidence regarding the effectiveness of mHealth interventions on hemoglobin A1C, body weight and blood glucose in immigrant populations.

This review found a promising trend of reduced anxiety, depression, and diabetes-related distress following mHealth interventions. This finding is comparable with a previous review, which reported that mHealth interventions have positive effects on depression and schizophrenia among patients with mental health problems [33]. Notably, research has shown that a considerable proportion of immigrant populations with diabetes experience elevated emotional burdens, and diabetes-related distress [34–36]. However, few mHealth interventions in the included studies incorporated components related to providing information and support for participants’ mental health. Future research should consider integrating mental health support into the design of mHealth interventions to offer more comprehensive care for immigrants with diabetes.

While mHealth interventions are widely advocated, previous research has indicated that many barriers impede clinical engagement with these interventions in healthcare settings, such as lack of equipment, technological and health illiteracy, and the high costs of accessing mHealth interventions [37, 38]. A recent study examining the use of mHealth interventions among 91 Chinese immigrants found that, despite high ownership rates of mobile devices, only 23% of participants (*n* = 21) reported using the internet to search for diabetes-related information in the past 12 months [39]. The barriers to utilizing mHealth interventions for diabetes management among immigrant populations are unclear, which warrants future research.

This review also found that few existing studies on mHealth interventions among immigrant populations employed a qualitative design. Compared to quantitative studies, qualitative inquiries can provide a rich and in-depth understanding of participants’ experiences with healthcare interventions. Future research could incorporate qualitative components or a mixed methods approach to obtain an in-depth understanding of the roles of mHealth interventions in helping immigrants manage their diabetes.

## Limitations and direction for future research

The findings of this review are primarily based on pilot studies with small sample sizes and limited statistical power, which reduces the conclusiveness of the results. In addition, this review includes only studies published in English, potentially missing relevant research in other languages. Due to the small number of studies available, as well as the variability in study designs, interventions, and immigrant groups, this review cannot provide definitive conclusions regarding the effectiveness of mHealth interventions on each healthcare outcome of diabetes. Therefore, there is a clear need for more rigorous research to generate robust evidence, enabling future meta-analyses to offer a more robust understanding of this field. The findings are limited to interventions exclusively designed for first-generation immigrants; studies that included immigrants as part of broader populations were not considered. Another limitation of this review is that MeSH terms were not used in the PubMed search strategy. Instead, the literature search relied on text words to obtain a comprehensive identification of relevant studies.

## Conclusion

To the best of our knowledge, this review is the first to summarize the current evidence of mHealth interventions specifically tailored to immigrant populations with diabetes. This review demonstrates the feasibility and acceptability of mHealth interventions for diabetes management among immigrant populations. The findings suggest that mHealth interventions are promising for improving health, psychosocial, and behavioral outcomes. The findings could provide valuable insights for future interventions and strategies to enhance diabetes care for immigrant populations. These findings may also contribute to reducing disparities in access to diabetes care among immigrant populations and could be generalizable to a broader range of immigrant communities. Future RCTs with larger sample sizes are needed to provide more robust evidence of the effectiveness of mHealth interventions. Importantly, this review highlights the overall scarcity of mHealth-related studies focused on immigrant populations with diabetes and calls for more research to determine how to best support this underserved group.

## Supplementary Information

Additional File 1: Supplementary Table 1: Search Strategy for Each Database. Supplementary Table 2: Methodological Quality of the Included Studies (*n* = 7). 

## Data Availability

The datasets used and/or analysed during the current study are available from the corresponding author on reasonable request.

## Abbreviations

US: United States
mHealth: Mobile Health
RCT: Randomized Controlled Trials
PRISMA: Preferred Reporting Items for Systematic Reviews and Meta-Analyses
PICO: Patient/Population, Intervention, Comparison, and Outcomes
MMAT: Mixed-Methods Appraisal Tool
