# Supplementary material for: Mobile health interventions tailored to immigrant populations with diabetes: an integrative review
Source: BMC Public Health. 2025 Nov 14;25:3954. doi: 10.1186/s12889-025-25241-3 (PMC12619219; doi:10.1186/s12889-025-25241-3)
Supplement: Supplementary file 1 — Additional File 1: Supplementary Table 1: Search Strategy for Each Database. Supplementary Table 2: Methodological Quality of the Included Studies (n = 7). [file 12889_2025_25241_MOESM1_ESM.docx]

**Additional File 1:**

Supplementary Table 1: Search Strategy for Each Database.

| **Database** | **Search strategy** | **Search results** |
| --- | --- | --- |
| PubMed | ((diabetes or diabetes mellitus) AND (telemedicine OR telehealth OR e-health OR eHealth OR mHealth OR mobile health OR digital health OR video OR message OR internet)) AND (migrant or immigrant) | 88 |
| Web of Science | diabetes or diabetes mellitus (All Fields) and telemedicine OR telehealth OR e-health OR eHealth OR mHealth OR mobile health OR digital health OR video OR message OR internet (All Fields) and migrant or immigrant (All Fields) | 100 |
| Cochrane Library | 158 Trials matching diabetes or diabetes mellitus in All Text AND telemedicine OR telehealth OR e-health OR eHealth OR mHealth OR mobile health OR digital health OR video OR message OR internet in All Text AND migrant or immigrant in All Text - (Word variations have been searched) | 158 |
| CINAHL Ultimate | AB (diabetes or diabetes mellitus or diabetic) AND TX ( telemedicine OR telehealth OR e-health OR eHealth OR mHealth OR mobile health OR digital health OR video OR message OR internet ) AND TX ( migrant or immigrant )  Expanders - Apply equivalent subjects  Search modes – Proximity | 504 |
| Embase | Embase <1947 to present>  1. (diabetes* or diabetes mellitus* or diabetic*).mp. [mp=title, abstract, heading word, drug trade name, original title, device manufacturer, drug manufacturer, device trade name, keyword heading word, floating subheading word, candidate term word]  2. (telemedicine or telehealth or e-health or eHealth or mHealth or mobile health or digital health or video or message or internet or tele* or mobile* or digital*).mp. [mp=title, abstract, heading word, drug trade name, original title, device manufacturer, drug manufacturer, device trade name, keyword heading word, floating subheading word, candidate term word]  3. (migrant* or immigrant*).mp. [mp=title, abstract, heading word, drug trade name, original title, device manufacturer, drug manufacturer, device trade name, keyword heading word, floating subheading word, candidate term word]  4. 1 and 2 and 3 | 185 |
| APA PsycInfo 1806 to July Week 4 2024 | APA PsycInfo <1806 to July Week 4 2024>  1. (diabetes or diabetes mellitus or diabetic).mp. [mp=title, abstract, heading word, table of contents, key concepts, original title, tests & measures, mesh word]  2. (migrant or immigrant).mp. [mp=title, abstract, heading word, table of contents, key concepts, original title, tests & measures, mesh word]  3. (telemedicine or telehealth or e-health or eHealth or mHealth or mobile health or digital health or video or message or internet).mp. [mp=title, abstract, heading word, table of contents, key concepts, original title, tests & measures, mesh word]  4. 1 and 2 and 3 | 12 |

Supplementary Table 2: Methodological Quality of the Included Studies (n = 7).

| **Study** | **Appraisal criteria** | | | | | | | |
| --- | --- | --- | --- | --- | --- | --- | --- | --- |
|  |  | **S1** | **S2** | **C1** | **C2** | **C3** | **C4** | **C5** |
| Kim  2009 | RCT  (pilot) | Yes | Yes | Yes | Yes | Yes | No | Yes |
| Kim  2015 | RCT | Yes | Yes | Yes | Yes | Yes | Yes | Yes |
| McElfish  2019 | Pre-post (pilot) | Yes | Yes | Yes | Yes | Yes | No | Yes |
| Rechenberg  2021 | RCT  (pilot) | Yes | Yes | Yes | Yes | Yes | Yes | Yes |
| Hu  2022 | Pre-post (pilot) | Yes | Yes | Yes | Yes | Yes | No | Yes |
| Hu  2024 | RCT  (pilot) | Yes | Yes | Yes | Yes | Yes | Yes | Yes |
| Shah  2024 | RCT | Yes | Yes | Yes | Yes | Yes | Yes | Yes |

Note: S = screening question; C = criteria; RCT = randomised controlled trial.
